# Computation Offloading and User-Clustering Game in Multi-Channel Cellular Networks for Mobile Edge Computing

**DOI:** 10.3390/s23031155

**Published:** 2023-01-19

**Authors:** Yan-Yun Huang, Pi-Chung Wang

**Affiliations:** Department of Computer Science and Engineering, National Chung Hsing University, Taichung 402, Taiwan

**Keywords:** computation offloading, clustering, game theory, Nash equilibrium, mobile edge computing

## Abstract

Mobile devices may use mobile edge computing to improve energy efficiency and responsiveness by offloading computation tasks to edge servers. However, the transmissions of mobile devices may result in interference that decreases the upload rate and prolongs transmission delay. Clustering has been shown as an effective approach to improve the transmission efficiency for dense devices, but there is no distributed algorithm for the optimization of clustering and computation offloading. In this work, we study the optimization problem of computation offloading to minimize the energy consumption of mobile devices in mobile edge computing by adaptively clustering devices to improve the transmission efficiency. To address the optimization problem in a distributed manner, the decision problem of clustering and computation offloading for mobile devices is formulated as a potential game. We introduce the construction of the potential game and show the existence of Nash equilibrium in the game with a finite enhancement ability. Then, we propose a distributed algorithm of clustering and computation offloading based on game theory. We conducted a simulation to evaluate the proposed algorithm. The numerical results from our simulation show that our algorithm can improve offloading efficiency for mobile devices in mobile edge computing by improving transmission efficiency. By offloading more tasks to edge servers, both the energy efficiency of mobile devices and the responsiveness of computation-intensive applications can be improved simultaneously.

## 1. Introduction

Various computation-intensive mobile applications, such as online gaming, machine learning, and virtual/augmented reality, have been developed. Some of these applications may have a delay-constraint requirement, but satisfying the requests of these applications from mobile devices (MDs) is difficult because these devices have only restricted resources [1]. Mobile edge computing has emerged as a crucial 5G technology. MEC servers are deployed at basestations in the close proximity to MDs to provide the capability of storage and computation for MDs [2]. This technology provides the benefits of improving transmission quality and efficient network operation [3].

Although mobile edge computing could improve the application performance for MDs, the simultaneous transmissions of MDs may degrade channel quality. As a result, the transmission performance is degraded, prolonging the response latency [4]. Although multi-channel communication can be applied to improve transmission performance by assigning MDs to different channels [5], the spectrum resources may still not be enough to accommodate all MDs [6].

To ease the communication overhead for MEC, it is possible to generate clusters of MDs, where only the cluster head of each cluster communicates with the basestation. In each cluster, the cluster members with computation offloadings transmit their data to the cluster head and the cluster head transmits data to the basestation on behalf of all MDs in the cluster. With the MD clustering, the transmission performance can be improved by allocating spectrum resources to cluster heads. However, the optimization problem of clustering and computation offloading for MDs has not been addressed in a distributed manner in the previous literature.

In this paper, we model the problem of clustering and computation offloading for MDs as a competitive game, where each MD attempts to minimize its overhead by independently adjusting its clustering and offloading decision until a Nash equilibrium is reach. As compared with a centralized approach, the competitive game enables a distributed model for better flexibility and scalability. We summarize the contributions of this work as below:

1. *Clustering and computation offloading for MDs*: To cope with the communication overhead caused by the interference among MDs with computation offloading, we propose a system model that clusters MDs, where only cluster heads can communicate with the basestation. Cluster members can forward their requests through their cluster heads to reduce the number of concurrent transmissions and improve the transmission performance.
2. *Formulation of a distributed competitive game*: Based on the system model, a potential game where each MD selfishly selects a decision to minimize its overhead is formulated. For the proposed game, we show the existence of Nash equilibrium, where no MD can achieve better performance by altering its strategy. With the existence of a Nash equilibrium, the game will converge within limited number of iterations.
3. *An algorithm of the distributed clustering and computation offloading game*: We developed an efficient algorithm, namely, distributed clustering and computation offloading (DCCO). Since the algorithm is performed in a distributed manner, the decision overhead is shared by all MDs within a network. Our algorithm is evaluated to show the performance improvement in terms of the number of successful computation offloadings, energy consumption and response delay. The convergence performance of the algorithm is also investigated.

The rest of this paper is organized as follows. Section 2 reviews related work. In Section 3, we present the system model of clustering and computation offloading. Then, we formulate the problem of clustering and computation offloading for MDs as a competitive game in Section 4, where the existence of Nash equilibrium is demonstrated. In Section 5, we present the proposed DCCO algorithm and explore the convergence of the DCCO game. To evaluate the performance of the proposed algorithm, we conducted experiments upon different algorithms and show their numerical results in Section 6. Finally, the conclusions of this paper are provided in Section 7.

## 2. Related Works

Researchers have exploited the technology of mobile edge computing for Internet of Things (IoT) and in the 5G framework for computation offloading. You et al. studied a near-optimal design with a threshold-based structure for network resource allocation in a multi-user mobile-edge computing offloading (MECO) system based on TDMA/OFDMA [7]. Malik et al. proposed parallel execution for computation tasks [8]. Guan et al. employed neighboring devices for cooperative partial offloading [9]. Zaman et al. incorporated machine learning for mobility prediction to improve resource efficiency [10,11]. The tradeoff between delay and energy consumption can also be addressed by an iterative search algorithm [12]. This algorithm yields the optimal solution in multi-device environments. The above algorithms are performed in a centralized manner for better optimization, but they may suffer from the cost of gathering statistical data from all MDs.

There are also implementations operated in a decentralized manner, where mobile devices make offloading decisions based on their own interests without forwarding statistics data to the MEC server. Tran et al. improved the performance gain of computation offloading for MEC with multiple servers [13]. Dinh et al. optimized the decisions of task allocation and CPU frequency with an offloading framework to minimize both total execution latency and energy consumption [14]. Bi et al. maximized the total computation rate of all wireless devices based on wireless power transfer for energy-harvesting wireless devices with a computation offloading policy [15]. Neto et al. presented a user-level online offloading framework [16] in which each device is equipped with a decision engine to minimize the remote-execution overhead. Chen et al. developed an online peer offloading structure by using the Lyapunov technique to handle spatially uneven computation workloads and prevent long computation latency in overloaded small-cell basestations [17]. Mazouzi et al. focused on computation offloading over a heterogeneous cloudlet environment for mobile devices whose tasks have different energy and latency constraints [18]. They proposed a heuristic approach of distributed linear relaxation based on the Lagrangian decomposition method.

There have also been studies applying game theory to solve the problem of computation offloading in a distributed framework. Yang et al. took a small cell network with multiple users and multiple MEC servers into account to design a threshold-based game-theoretic approach [19]. Guo et al. introduced a hybrid fiber-wireless (FiWi) network to support a system of integrating a centralized cloud and multi-access edge computing [20]. To address the collaborative computation offloading problem and overcome the drawback of centralized management, an approximation greedy strategy and a game theory as a distributed scheme was developed. He et al. investigated the edge-user-allocation problem from the perspectives of application vendors in edge computing, where users can make their own allocation decisions [21].

According to the previous literature [22], we are aware that one factor affecting the performance of computation offloading is the communication between mobile devices and MEC servers. Channel management was considered in the previous studies of communication. Li et al. investigated the problems of radio and computational resources to improve spectrum efficiency for both static and dynamic tasks from mobile devices for vehicular edge computing [23]. Ning et al. proposed a hybrid computation offloading structure for real-time interactive systems [24]. They formulated a joint problem of task offloading, sub-channel assignment and power allocation. Alsen et al. introduced an MEC system for unmanned aerial vehicles with the optimization problem of task offloading. They also considered bandwidth allocation for IoT devices and resource allocation in local computation [25]. Tun et al. presented a system of virtualized multi-access edge computing, where network bandwidth and computation resource are sliced [26]. Cheng et al. aimed at a multi-user and multi-MEC server scenario based on OFDMA [27]. They jointly investigated task offloading policies and radio resource allocation. In this work, we also use OFDMA for the communications between MDs and basestations.

Clustering is a technique to categorize entities into different groups, and the entities of each group share a certain level of similarity [28]. Numerous approaches have been proposed to address the optimization of clustering [29,30]. Although the technique of clustering is applied to wireless transmission, previous studies of computation offloading rarely exploited the concept of clustering to improve the performance of offloading based on game theory. Hong et al. declared a robust optimization algorithm of energy and power with fault tolerance to overcome diverse electrical power and computing strength [31]. Attiah et al. implemented a game-theoretical clustering framework to allow for the differentiation of a cluster head’s obligation between each node to manage energy usage [32]. Afsar et al. aimed at the issue of energy shortage by proposing the splitting network and game-theory-based clustering algorithm [33]. Loomba et al. presented the development of an energy-efficient stochastic leader-selection algorithm [34], which uses the distance between mobile devices and the basestation to select the best cluster head as offloading agent. The algorithm also improves the energy usage of the interaction between mobile handsets and an application server. Bouet et al. used a MEC-clustering algorithm to consolidate edge communications [35]. Du et al. introduced a fast and self-adaptive clustering algorithm to obtain a accurate density threshold by optimizing the sum of squared errors formula with limited iterations [36]. He et al. offloaded computational tasks in MEC-based ultra dense networks from the core network by the perception of AP-clustering [37].

To our knowledge, there is no distributed clustering algorithm for MEC computation offloading. We are thus motivated to combine the decision problems of clustering and computation offloading as a competitive game and propose an efficient algorithm.

## 3. System Model

**Figure 1:**
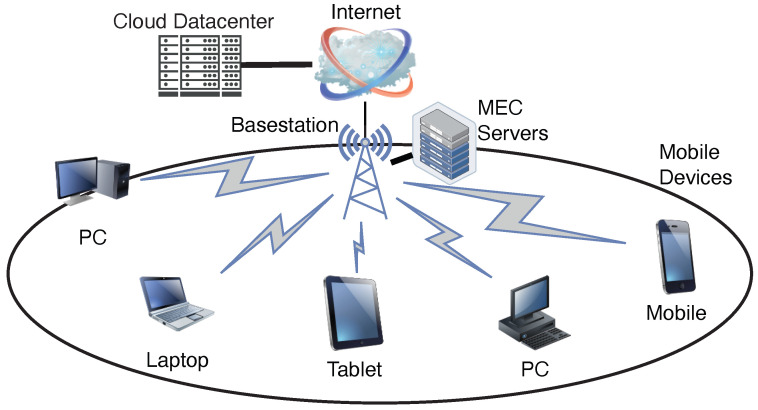
An illustration of mobile edge computing.

In this section, we show the model for the distributed clustering and computation offloading game. In the model, a set of mobile devices expressed as N={1,2…,n} are randomly distributed in a cell. These mobile devices have computation tasks, which must be completed with a limited delay. Each basestation is connected to a MEC server with computational capabilities, as shown in Figure 1. The computation tasks which cannot be accommodated by MEC servers will be forwarded to remote cloud datacenter, but the computation tasks offloaded to the datacenter may suffer from long latency. In this work, we consider a static scenario where the states of mobile devices do not change during the operation of computation offloading.

**Figure 2:**
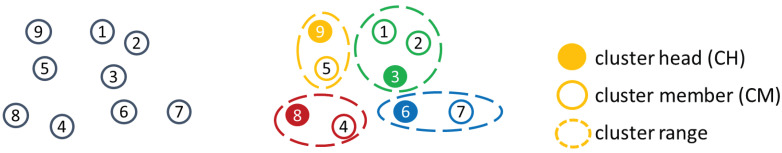
An example of MD clustering.

We further propose a clustering approach which incorporates short-distance communication, e.g., Wi-Fi in WLAN or dedicated short-range communications (DSRC) in VANET, to minimize communication overheads. As shown in the left of Figure 2, mobile devices are randomly distributed in a cell, where each device has a unique identifier. Within a range of short-distance communication, a mobile device can select a nearby MD as its cluster head, where the cluster heads are denoted by solid circles in the middle of Figure 2. The cluster heads collect data from their members of the same cluster and transmit the data to the basestation. As a result, the cluster members do not directly communicate with the basestation to reduce interference.

The node clustering generates a set of clusters denoted as $CL=\{cl_{1},cl_{2}\ldots ,cl_{t}\}$, 1≤t≤N. With the clusters, a cluster head could achieve a superior transmission rate to the basestation and shorten the transmission latency. In this work, we neglect the occurrence of collisions. If the collision ratios are too high to enable clustering, our scheme can still opt for direct communications between mobile devices and the basestation. We also assume that data from the computation task of each MD would be encrypted before the transmission to avoid the concern of privacy breaches.

In the following subsections, we describe the system model in detail. Section 3.1 describes the communication model. The computation model is introduced in Section 3.2. The notation used in both subsections is listed in Table 1.

### 3.1. Communication Model

In this section, we describe the proposed communication model for mobile edge computing. Each basestation has a set of wireless channels represented as M={1,2…,m}. MDs share these channels by using OFDMA to assign a subset of channel resources. We denote $off_{n}\in \left\{0\right\}\cup M,c_{n}\in \left\{0\right\}\cup CL$ as the channel and clustering selection of each MD *n*, respectively. With the decision profiles, $OFF=\{off_{1},off_{2}\ldots ,off_{n}\}$ and $C=\{c_{1},c_{2}\ldots ,c_{n}\}$, of all mobile devices, we can compute the uplink data transmission rate of MD *n* which opts to offload via the MD clustering approach by using the following formula:

(1) $$r_{n}\left(off,c\right)=Wlog_{2}\left(1+\frac{p_{n}g_{n,s}}{\omega_{0}+\sum_{m\in N:off_{m}=off_{n},c_{m}\neq c_{n},m\neq n}p_{m}g_{m,s}}\right)$$

*W* is the channel bandwidth, and $p_{n}$ is the transmission power of MD *n*. $g_{n,s}$ denotes the channel gain between the MD *n* and the basestation *s* according to the path loss and shadowing. $\omega_{0}$ denotes the background noise power.

From Equation (1), we can recognize that mobile devices may incur heavy interference when numerous devices select the same wireless channel. By clustering mobile devices to reduce excessive transmissions in a wireless channel, the uplink transmission rate can be improved.

### 3.2. Computation Model

Next, we present the computation model. We assume that each mobile device *n* has a computational task, $J_{n}=\left\{B_{n},D_{n}\right\}$, to be executed either locally on MD *n*, or offloaded to MEC server based on its decision. $B_{n}$ indicates the data size of computation input to offload, and $D_{n}$ denotes the total number of CPU cycles required to accomplish $J_{n}$. Then, we can calculate the computation overhead in terms of delay and energy consumption for local computing and edge computing.

#### 3.2.1. Local Computing

Let $f_{n}^{l}$ be the computational capability of MD *n*, i.e., the number of CPU cycles per second. If MD *n* calculates its task $J_{n}$ locally, the delay is $T_{n}^{l}=\frac{D_{n}}{f_{n}^{l}}$, and the energy consumption can be formulated as:

(2) $$E_{n}^{l}=D_{n}\varepsilon_{n}^{l},$$

where $\varepsilon_{n}^{l}$ is the coefficient of the consumed energy per CPU cycle.

#### 3.2.2. Edge Computing

The main difference between edge and local computing is the additional overheads for the computation offloading of a MD. The overhead includes the offloading delay and energy consumption for transmitting data to the MEC server. The delay and energy consumption for the offloading are defined as $T_{n}^{off}$ and $E_{n}^{off}$, where $E_{n}^{off}=T_{n}^{off}p_{n}$. After MD *n* finishes the transmission, the MEC server performs the computation task $J_{n}$ remotely. Thus, the delay for executing task $J_{n}$ is $T_{n}^{e}$. We ignore the energy consumption of the MEC server.

There are two approaches to offload the task of each MD, where a MD can either directly offload its task to the MEC server or upload its data through the cluster head. In the later case, the cluster head transmits the data of all cluster members based on the following equation:

(3) $$B_{n}^{cl_{n}}=\sum_{n\in cl_{n}}B_{n}$$

Let $f^{e}$ be the computational capability of the MEC server. If MD *n* offloads its task $J_{n}$ to the MEC server through the basestation, the total delay and the energy consumption can be expressed as:

(4) $$\begin{array}{ccc} T_{n}^{e} & = & T_{n}^{off}+T_{n}^{exe}=\frac{B_{n}}{r_{n}\left(off,c\right)}+\frac{D_{n}}{f^{e}} \end{array}$$

(5) $$\begin{array}{ccc} E_{n}^{e} & = & E_{n}^{c}=\frac{p_{n}B_{n}}{r_{n}\left(off,c\right)} \end{array}$$

The possible values of $B_{n}$ are listed in Equation (6), and those of $p_{n}$ are listed in Equation (7).

(6) $$\begin{array}{ccc} B_{n} & = & \left\{\begin{array}{cc} B_{n}, & \mathrm{MD}\;n\;\mathrm{as}\;\mathrm{an}\;\mathrm{offloader}\\ B_{n}^{cl_{n}}, & \mathrm{MD}\;n\;\mathrm{as}\;a\;\mathrm{cluster}\;\mathrm{head}\\ 0, & \mathrm{MD}\;n\;\mathrm{as}\;a\;\mathrm{cluster}\;\mathrm{member} \end{array}\right. \end{array}$$

(7) $$\begin{array}{ccc} p_{n} & = & \left\{\begin{array}{cc} p_{n}^{e}, & \mathrm{MD}\;n\;\mathrm{as}\;\mathrm{an}\;\mathrm{offloader}\;\mathrm{or}\;a\;\mathrm{cluster}\;\mathrm{head}\\ p_{n}^{w}, & \mathrm{MD}\;n\;\mathrm{as}\;a\;\mathrm{cluster}\;\mathrm{member} \end{array}\right. \end{array}$$

We further combine Equations (2) and (5) to obtain computation overhead of clustering-based offloading for each MD:

(8) $$E_{n}=\left\{\begin{array}{cc} E_{n}^{l}, & \mathrm{if}\;c_{n}=0\;\mathrm{and}\;off_{n}=0\\ E_{n}^{e}, & \mathrm{if}\;c_{n}=0\;\mathrm{and}\;off_{n}>0\\ E_{n}^{c}, & \mathrm{if}\;c_{n}>0\;\mathrm{and}\;off_{n}>0 \end{array}\right.$$

We do not consider the downlink transmission of results in the model, because the result size is usually much smaller than the input data [19].

## 4. Problem Formulation

Next, we formulate the clustering and computation offloading problem for mobile edge computing based on the proposed communication and computation model.

### 4.1. Game Formulation

We denote $s_{n}=\left\{off_{n},c_{n}\right\}$ as the channel and clustering decisions of the mobile device *n*. Let $s_{-n}=\left\{s_{1},\ldots ,s_{n-1},s_{n+1},\ldots ,s_{N}\right\}$ be the decisions of the other MDs and $-s_{n}$ be decisions not chosen by mobile device *n*. Given the decisions of the other MDs, $s_{-n}$, mobile device *n* chooses the decision $s_{n}$ as the solution of the following equation:

(9) $$\begin{array}{ccc} \mathrm{OMIN} & : & min\sum_{n\in N}E_{n}\left(s_{n},s_{-n}\right)\\ C_{1} & : & {\mathrm{off}}_{n}\in \left\{0,1,2\ldots ,M\right\},\\ C_{2} & : & c_{n}\in \left\{0,cl_{1},cl_{2}\ldots ,cl_{t}\right\},\\ C_{3} & : & D_{n}\left(s_{n}\right)I_{\left\{{\mathrm{off}}_{n}\neq 0,c_{n}\neq 0\right\}}\leq D_{n}\left(-s_{n}\right)I_{\left\{{\mathrm{off}}_{n}\neq 0,c_{n}\neq 0\right\}}, \end{array}$$

where $C_{1}$ and $C_{2}$ are defined in Section 3.1. $I_{\left\{S\right\}}$ is used to indicate whether a decision variable is true. For example, if *S* is true, $I_{\left\{S\right\}}=1$; otherwise, $I_{\left\{S\right\}}=0$.

According to Equation (8), we can set the problem of computation energy consumption as the following equation:

(10) $$E_{n}\left(s_{n},s_{-n}\right)=\left\{\begin{array}{cc} E_{n}^{l}, & \mathrm{if}\;c_{n}=0\;\mathrm{and}\;off_{n}=0\\ E_{n}^{e}, & \mathrm{if}\;c_{n}=0\;\mathrm{and}\;off_{n}>0\\ E_{n}^{c}, & \mathrm{if}\;c_{n}>0\;\mathrm{and}\;off_{n}>0 \end{array}\right.$$

Then, we formulate the overhead minimization problem (OMIN) to be a game of strategy, $\Gamma =(N,{\left\{S_{n}\right\}}_{n\in N},{\left\{E_{n}\right\}}_{n\in N})$, where the players are the mobile devices ∈N. $S_{n}$ is the set of strategies including offloading ${\mathrm{off}}_{n}$ and clustering decision $c_{n}$ for player *n*. The energy consumption $E_{n}\left(s_{n},s_{-n}\right)$ of each MD is the expense function to be minimized for player *n*. Consequently, we regard the game as the multi-MD clustering and computation offloading game and show the existence of a Nash equilibrium in the game.

**Definition** **1.** *A set of strategies $s^{★}=\left\{s_{1}^{★},\ldots ,s_{n}^{★}\right\}$ reaches a Nash equilibrium for the clustering and computation offloading game with multiple MDs where no MD can additionally reduce its energy consumption by adjusting its strategy in the equilibrium $s^{★}$; i.e.,*

(11) $$E_{n}\left(s_{n}^{★},s_{-n}^{★}\right)<E_{n}\left(s_{n},s_{-n}^{★}\right),\forall s_{n}\in S_{n},n\in N.$$

Based on the above definition, when the game of clustering and computation offloading reaches a Nash equilibrium, each MD has a mutually satisfactory solution.

### 4.2. Nash Equilibrium of the DCCO Game

Next, we show the presence of Nash equilibrium for the clustering and computation offloading (DCCO) game.

**Definition** **2.** *It can be inferred from a potential game that a potential function Φ(s) is needed so that $\forall n\in N,s_{n},s_{n}^{★}\in S_{n},s_{-n}\in S_{-n}$:*

(12) $$\begin{array}{cc} \; & E_{n}\left(s_{n}^{★},s_{-n}\right)-E_{n}\left(s_{n},s_{-n}\right)<0\\ \Rightarrow & \Phi_{n}\left(s_{n}^{★},s_{-n}\right)-\Phi_{n}\left(s_{n},s_{-n}\right)<0. \end{array}$$

**Lemma** **1.** *Given a set of strategies with offloading and clustering decisions s={off,c}, edge computing with clustering is favorable if its received interference from other MDs, $\tau_{n}\left(s\right)=\tau_{n}\left(off,c\right)=\sum_{i\in N\setminus \left\{n\right\}:off_{i}=off_{n},c_{i}\neq c_{n}}p_{i}g_{i,s}$, in the channel, satisfies that $\tau_{n}\left(s\right)\leq \Theta_{n}$, where the threshold is expressed as*

$$\Theta_{n}=\frac{p_{n}g_{n,s}}{\frac{p_{n}B_{n}}{2^{WE_{n}^{l}}-1}}-\omega_{0}$$

**Proof.**  According to Equations (2) and (5), if we anticipate the edge computing with clustering is advantageous compared with local computation for MD *n*, the following circumstance, $E_{n}^{c}\left(s\right)\leq E_{n}^{l}\left(s\right)$, must occur; i.e., $\frac{B_{n}}{r_{n}\left(s\right)}p_{n}\leq E_{n}^{l}$. Therefore, we can derive the following equation:

$$r_{n}\left(s\right)\geq \frac{B_{n}}{E_{n}^{l}}p_{n}.$$

Based on Equation (1), we can get that

(13) $$\sum_{i\in N\setminus \left\{n\right\}:off_{i}=off_{n},c_{i}\neq c_{n}}p_{i}g_{i,s}\leq \frac{p_{n}g_{n,s}}{\frac{p_{n}B_{n}}{2^{WE_{n}^{l}}-1}}-\omega_{0}.$$

   □

The condition after ‘∖’ indicates the exception cases.

Through Lemma 1, we notice that if a MD receives low interference in its channel, the MD will prefer to upload its task to the MEC server via its cluster head. In contrast, if the obtained interference from other MDs on the same channel is high, the MD should perform its task locally.

**Theorem** **1.** *The clustering and computation offloading game is a possible game to reach a Nash equilibrium with a finite enhancement ability.*

**Proof.**  First, we assume that s={off,c} are the channel and clustering decisions of mobile devices. The potential equation for clustering and computation offloading game can be presented as

(14) $$\Phi \left(s\right)=\frac{1}{2}\sum_{i\in S}\sum_{j\in S\setminus \{i\}}p_{i}g_{i,s}p_{j}g_{j,s}I_{\left\{{off}_{i}={off}_{j},c_{i}\neq c_{j}\right\}}I_{\left\{c_{i}>0\right\}}+\sum_{i\in S}p_{i}g_{i,s}\Theta_{i}I_{\left\{{off}_{i}=0,c_{i}=0\right\}}$$

   □

Equation (14) can be expressed as the following:

(15) $$\begin{array}{ccc} \Phi (s) & = & \frac{1}{2}\sum_{j\in S\setminus \{k\}}p_{k}g_{k,s}p_{j}g_{j,s}I_{\left\{{off}_{j}={off}_{k},c_{j}\neq c_{k}\right\}}I_{\left\{c_{k}>0\right\}}\\ & & +\frac{1}{2}\sum_{i\in S\setminus \{k\}}p_{i}g_{i,s}p_{k}g_{k,s}I_{\left\{{off}_{k}={off}_{i},c_{k}\neq c_{i}\right\}}I_{\left\{c_{i}>0\right\}}\\ & & +\frac{1}{2}\sum_{i\in S\setminus \{k\}}\sum_{j\in S\setminus \{i,k\}}p_{i}g_{i,s}p_{j}g_{j,s}I_{\left\{{off}_{i}={off}_{j},c_{i}\neq c_{j}\right\}}I_{\left\{c_{i}>0\right\}}\\ & & +p_{k}g_{k,s}\Theta_{k}I_{\left\{{off}_{k}=0,c_{k}=0\right\}}\\ & & +\sum_{i\in S\setminus \{k\}}p_{i}g_{i,s}\Theta_{i}I_{\left\{{off}_{i}=0,c_{i}=0\right\}} \end{array}$$

We can get the following equation:

(16) $$\begin{array}{ccc} & & \sum_{j\in S\setminus \{k\}}p_{k}g_{k,s}p_{j}g_{j,s}I_{\left\{{off}_{j}={off}_{k},c_{j}\neq c_{k}\right\}}I_{\left\{c_{k}>0\right\}}\\ & = & \sum_{i\in S\setminus \{k\}}p_{i}g_{i,s}p_{k}g_{k,s}I_{\left\{{off}_{k}={off}_{j},c_{k}\neq c_{j}\right\}}I_{\left\{{off}_{i}>0,c_{i}>0\right\}} \end{array}$$

Let $\Xi (s_{S\setminus \{k\}})$ be the following equation:

(17) $$\frac{1}{2}\sum_{i\in S\setminus \{k\}}\sum_{j\in S\setminus \{i,k\}}p_{i}g_{i,s}p_{j}g_{j,s}I_{\left\{{off}_{i}={off}_{j},c_{i}\neq c_{j}\right\}}I_{\left\{c_{i}>0\right\}}+\sum_{i\in S\setminus \{k\}}p_{i}g_{i,s}\Theta_{i}I_{\left\{{off}_{i}=0,c_{i}=0\right\}}.$$

With Equations (15) and (16), we can obtain the following equation:

(18) $$\Phi \left(s\right)=\sum_{j\in S\setminus \{k\}}p_{i}g_{i,s}p_{k}g_{k,s}I_{\left\{{off}_{k}={off}_{i},c_{k}\neq c_{i}\right\}}I_{\left\{c_{i}>0\right\}}+p_{k}g_{k,s}\Theta_{k}I_{\left\{{off}_{k}=0,c_{k}=0\right\}}+\Xi \left(s_{S\setminus \{k\}}\right),$$

where $\Xi (s_{S\setminus \{k\}})$ is self-reliant for MD *k*’s strategy $s_{k}$. With Equation (13), we obtain the following equation:

(19) $$E_{k}\left(s\right)=\sum_{j\in S\setminus \{k\}}p_{j}g_{j,s}I_{\left\{{off}_{j}={off}_{k},c_{j}\neq c_{k}\right\}}I_{\left\{c_{k}>0\right\}}+\Theta_{k}I_{\left\{{off}_{k}=0,c_{k}=0\right\}}$$

Then, we assume that MD *k* will have the situation $E_{k}\left(s_{k}^{★},s_{-k}\right)<E_{k}\left(s_{k},s_{-k}\right)$, when it prefers updating its clustering and offloading decisions to cut down its cost. With the potential game’s definition, the request of updating would result in the situation $\Phi_{k}\left(s_{k}^{★},s_{-k}\right)<\Phi_{k}\left(s_{k},s_{-k}\right)$. We consider three cases—(1) $c_{k}>0,c_{k}^{★}>0$, (2) $c_{k}=0,c_{k}^{★}>0$ and (3) $c_{k}>0,c_{k}^{★}=0$—to analyze whether the convergence can be achieved for a given channel decision $off_{k}$.

Case 1 appears when the MD *k*’s clustering and offloading decisions are updated from the clustering $c_{k}>0$ toward the other clustering $c_{k}^{★}>0$. Based on Equation (1), it is obvious that the function $Wlog_{2}\alpha$ consistently grows by α, and the circumstance $E_{k}\left(s_{k}^{★},s_{-k}\right)<E_{k}\left(s_{k},s_{-k}\right)$ is known. We can get the result of the following equation:

(20) $$\sum_{i\in N\setminus \left\{k\right\}:{off}_{i}={off}_{k}^{★},c_{i}\neq c_{k}}p_{i}g_{i,s}<\sum_{i\in N\setminus \left\{k\right\}:{off}_{i}={off}_{k},c_{i}\neq c_{k}}p_{i}g_{i,s}$$

With Equations (18) and (20), we can get:

(21) $$\begin{array}{ccc} \Phi_{k}\left(s_{k},s_{-k}\right)-\Phi_{k}\left(s_{k}^{★},s_{-k}\right) & = & p_{k}g_{k,s}\sum_{i\in S\setminus \{k\}}p_{i}g_{i,s}I_{\left\{{off}_{k}={off}_{i},c_{k}\neq c_{i}\right\}}I_{\left\{c_{k}>0\right\}}\\ & & -p_{k}g_{k,s}\sum_{i\in S\setminus \{k\}}p_{i}g_{i,s}I_{\left\{{off}_{k}^{★}={off}_{i},c_{k}\neq c_{i}\right\}}I_{\left\{c_{k}>0\right\}}>0 \end{array}$$

Case 2 occurs when MD *k*’s decision is updated from a clustering decision $c_{k}=0$, i.e., no clustering, to another cluster $c_{k}^{★}>0$. The interference in the cluster $c_{k}^{★}>0$ should be smaller than the threshold of interference, i.e., $\sum_{i\in N\setminus \left\{n\right\}:{off}_{i}={off}_{k}^{★},c_{i}\neq c_{k}}p_{i}g_{i,s}<\Theta_{k}$ and $E_{k}\left(s_{k}^{★},s_{-k}\right)<E_{k}\left(s_{k},s_{-k}\right)$. The following equation can be yielded:

(22) $$\begin{array}{ccc} \Phi_{k}\left(s_{k},s_{-k}\right)-\Phi_{k}\left(s_{k}^{★},s_{-k}\right) & = & p_{k}g_{k,s}\Theta_{k}I_{\left\{{off}_{k}=0,c_{k}=0\right\}}\\ & & -p_{k}g_{k,s}\sum_{i\in S\setminus \{k\}}p_{i}g_{i,s}I_{\left\{{off}_{k}^{★}={off}_{i},c_{k}\neq c_{i}\right\}}I_{\left\{c_{k}>0\right\}}>0 \end{array}$$

The last case takes place when MD *k*’s clustering and offloading decisions are updated with the cluster $c_{k}>0$ toward the other clustering decision $c_{k}^{★}=0$, i.e., no clustering. As for $E_{k}\left(s_{k}^{★},s_{-k}\right)<E_{k}\left(s_{k},s_{-k}\right)$ and $\sum_{i\in N\setminus \left\{n\right\}:{off}_{i}={off}_{k}^{★},c_{i}\neq c_{k}}p_{i}g_{i,s}>\Theta_{k}$, we can generate the following equation:

(23) $$\begin{array}{ccc} \Phi_{k}\left(s_{k},s_{-k}\right)-\Phi_{k}\left(s_{k}^{★},s_{-k}\right) & = & p_{k}g_{k,s}\sum_{i\in S\setminus \{k\}}p_{i}g_{i,s}I_{\left\{{off}_{k}={off}_{i},c_{k}\neq c_{i}\right\}}I_{\left\{c_{k}>0\right\}}\\ & & -p_{k}g_{k,s}\Theta_{k}I_{\left\{{off}_{k}^{★}=0,c_{k}=0\right\}}>0 \end{array}$$

Based on the analysis for three cases of updating decisions, we can observe that a potential game of clustering and computation offloading can be formed to reach Nash equilibrium.

## 5. Distributed Clustering and Computation Offloading Game

In this section, we develop an efficient distributed clustering and computation offloading game among multiple MDs to achieve Nash equilibrium and analyze the convergence of the DCCO game.

### 5.1. Algorithm

The proposed algorithm is conducted in an iterative manner, as listed in Algorithm 1. Initially, the decisions of all MDs are local computation and without joining any clustering; i.e., $off_{n}=0,c_{n}=0$. In order to examine the interference information of different channels, each MD, say *n*, may transmit its offloading and clustering decisions to the basestation through the channel access. Then, the basestation will return the information of wireless channels and the locations of nearby mobile devices to MD *n*. The MD *n* can thus measure the interference of different channels to avoid poor transmission quality among MDs of the same channel in the previous iteration. The locations of nearby MDs can be used to identify the neighboring nodes. Once a MD *n* receives the feedback from the basestation, it will calculate the best solution, $\Delta_{n}\left(t\right)$, based on the OMIN problem, where each MD *n* independently selects its decisions of offloading $off_{n}$ and clustering $c_{n}$. If the decisions of current iteration are different to those of the previous one, i.e., $\Delta_{n}\left(t\right)\neq \Delta_{n}\left(t-1\right)$, the MD *n* transmits a request to the MEC server to compete for the permission to update its decision. Otherwise, the MD *n* does not forward any message to the MEC server to maintain its decision in this iteration; i.e., $off_{n}\left(t+1\right)=off_{n}\left(t\right),c_{n}\left(t+1\right)=c_{n}\left(t\right)$.
**Algorithm 1:**DCCO game.1:**Initialize:**offloading decision $off_{n}\left(0\right)=0$;clustering decision $c_{n}\left(0\right)=0$.2:**while** update decision request ≠∅ **do**3:   **for** each iteration *t* **do**4:     **for** each MD *n* **do**5:        Send a wireless signal toward basestation.6:        Receive information of channels and near MDs.7:        $\Delta_{n}\left(t\right)⟵$ compute best solution by (OMIN).8:     **end for**9:     **if** $\Delta_{n}\left(t\right)\neq \Delta_{n}\left(t-1\right)$ **then**10:        Send a request to update decision to MEC server.11:        **if** a permission of updating decision received from the MEC server **then**12:          Update offloading and clustering decisions          $off_{n}\left(t+1\right),c_{n}\left(t+1\right)=\Delta_{n}\left(t\right)$.13:        **else**14:          Keep offloading and clustering decisions          $off_{n}\left(t+1\right)=off_{n}\left(t\right),c_{n}\left(t+1\right)=c_{n}\left(t\right)$.15:        **end if**16:     **else**17:        Keep offloading and clustering decisions        $off_{n}\left(t+1\right)=off_{n}\left(t\right),c_{n}\left(t+1\right)=c_{n}\left(t\right)$.18:     **end if**19:   **end for**20:**end while**

When the MEC server receives update requests from MDs, the MEC server will randomly select and accept one of those requests and inform all MDs. Only the selected MD can update its decision, i.e., $off_{n}\left(t+1\right),c_{n}\left(t+1\right)=\Delta_{n}\left(t\right)$, and the other MDs keep their decisions unchanged as in the previous iteration—i.e., $off_{n}\left(t+1\right)=off_{n}\left(t\right),c_{n}\left(t+1\right)=c_{n}\left(t\right)$. The game will execute continuously until no update messages are transmitted to the MEC server. In other words, no mobile device can further reduce energy consumption by updating its decision. Therefore, the MEC server with the basestation will commit the decisions in the last iteration of the DCCO game, and each MD will perform its computational task based on its clustering and offloading decisions.

We note that it is possible that an MD may disconnect before convergence. In this case, the MD will no longer receive any updates to yield the final decision for computation offloading. The other MDs will ignore this MD and proceed to reach Nash equilibrium.

### 5.2. Convergence Analysis

Owing to Theorem 1, the algorithm of DCCO game will retain a Nash equilibrium within the limited iterations. In this subsection, we analyze the computational complexity of the DCCO algorithm.

Assume *F* iterations are required to finish the algorithm of the DCCO game for *N* mobile devices; the overall complexity of the DCCO game is O(FNlogN). We let $\Theta_{max}=max_{n\in N}\Theta_{n}$, $\Lambda_{n}=p_{n}g_{n,s}$, $\Lambda_{max}=max_{n\in N}\Lambda_{n}$ and $\Lambda_{min}=min_{n\in N}\Lambda_{n}$ and consider *F* iterations to reach convergence for the DCCO game. The following result can be obtained.

**Theorem** **2.** *DCCO game will end within at most $\frac{\Lambda_{max}^{2}}{2\Lambda_{min}}N^{2}+\frac{\Theta_{max}\Lambda_{max}}{\Lambda_{min}}N$ iterations when $\Theta_{n}$ and $\Lambda_{n}$ are both positive integers, ∀n∈N. That is, $F\leq \frac{\Lambda_{max}^{2}}{2\Lambda_{min}}N^{2}+\frac{\Theta_{max}\Lambda_{max}}{\Lambda_{min}}N$.*

**Proof.**  Based on Equation (14), we can obtain:

(24) $$0\leq \Phi \left(s\right)\leq \frac{1}{2}\sum_{i\in N}\sum_{j\in N}\Lambda_{max}^{2}+\sum_{i\in N}\Lambda_{max}\Theta_{max}=\frac{1}{2}\Lambda_{max}^{2}N^{2}+\Lambda_{max}\Theta_{max}N\;$$

□

We assume that a MD k∈N receives permission from the MEC server to update its current clustering decision from $c_{k}$ to another clustering decision $c_{k}^{★}$, in order to yield a decline in its expense function. According to Definition 2, the above condition also yields a decline in the potential function as well; i.e.,

(25) $$\Phi_{k}\left(s_{k}^{★},s_{-k}\right)>\Phi_{k}\left(s_{k},s_{-k}\right)+\Lambda_{min}.$$

Now, we discuss each of the three cases presented in Section 4.2: (1) $c_{k}>0,c_{k}^{★}>0$, (2) $c_{k}=0,c_{k}^{★}>0$ and (3) $c_{k}>0,c_{k}^{★}=0$.

Case 1: Based on Equation (21), we can obtain:

(26) $$\begin{array}{ccc} \Phi_{k}(s_{k},s_{-k})-\Phi_{n}\left(s_{k}^{★},s_{-k}\right) & = & \Lambda_{k}(\sum_{i\in S\setminus \{k\}}\Lambda_{i}I_{\left\{{off}_{k}={off}_{i},c_{k}\neq c_{i}\right\}}I_{\left\{c_{k}>0\right\}}\\ & & -\sum_{i\in S\setminus \{k\}}\Lambda_{i}I_{\left\{{off}_{k}^{★}={off}_{i},c_{k}\neq c_{i}\right\}}I_{\left\{c_{k}>0\right\}})>0. \end{array}$$

Due to the property of integers as $\Lambda_{i}$ for each MD in *N*,

(27) $$\sum_{i\in S\setminus \{k\}}\Lambda_{i}I_{\left\{{off}_{k}={off}_{i},c_{k}\neq c_{i}\right\}}I_{\left\{c_{k}>0\right\}}\geq \sum_{i\in S\setminus \{k\}}\Lambda_{i}I_{\left\{{off}_{k}^{★}={off}_{i},c_{k}\neq c_{i}\right\}}I_{\left\{c_{k}>0\right\}}+1$$

As a result, based on Equation (26), we can yield the following equation for Case 1:

$$\Phi_{k}\left(s_{k},s_{-k}\right)\geq \Phi_{k}\left(s_{k}^{★},s_{-k}\right)+\Lambda_{k}\geq \Phi_{k}\left(s_{k}^{★},s_{-k}\right)+\Lambda_{min}$$

Case 2: Based on Equation (22), we have the following equation:

(28) $$\Phi_{k}\left(s_{k},s_{-k}\right)-\Phi_{n}\left(s_{k}^{★},s_{-k}\right)=\Lambda_{k}\left(\Theta_{k}-\sum_{i\in S\setminus \{k\}}\Lambda_{i}I_{\left\{{off}_{k}={off}_{i},c_{k}\neq c_{i}\right\}}I_{\left\{c_{k}>0\right\}}\right)>0$$

Thus, based on Equation (28), Case 2 can be held by:

$$\Phi_{k}\left(s_{k},s_{-k}\right)\geq \Phi_{k}\left(s_{k}^{★},s_{-k}\right)+\Lambda_{k}\geq \Phi_{k}\left(s_{k}^{★},s_{-k}\right)+\Lambda_{min}$$

Case 3: Through the equivalent statements on Cases 1 and 2, we can demonstrate the following equation for the last case:

$$\Phi_{k}\left(s_{k},s_{-k}\right)\geq \Phi_{k}\left(s_{k}^{★},s_{-k}\right)+\Lambda_{min}$$

Hence, according to Equations (24) and (25) and by applying the potential function into a minimum state, the algorithm of the DCCO game will finish within $\frac{\Lambda_{max}^{2}}{2\Lambda_{min}}N^{2}+\frac{\Theta_{max}\Lambda_{max}}{\Lambda_{min}}N$ iterations, at most.

We consider that transmission power and channel gain of MD *n* in reality are both positive integers, i.e., $p_{n}\geq 0,g_{n,s}\geq 0$. Moreover, the condition of $\Theta_{n}\geq 0$ is non-negative to imply that the probability of MD *n* can achieve advantageous edge computing as compared with local computing. Our simulation results in Section 6 also demonstrate that the DCCO game can converge rapidly.

## 6. Simulation Results

In this section, we validate the effectiveness of the proposed algorithm based on simulations. We developed a Python-based simulator to perform our simulations. The simulation scenario included a basestation whose coverage was 100 m. There were 30 to 70 MDs randomly distributed in the cell. There were five channels, namely, M=5; the bandwidth of each channel was 5 MHz. The transmission power *p* was 150 mWatts. We set the path loss factor as 4. The data generated by MDs were 5000 kbits, and the number of required CPU cycles for computation tasks was $1000\times 10^{8}$. The computational capacity of the MEC server was $10\times 10^{9}$ cycles/s, and that of a MD was $1.0\times 10^{9}$ cycles/s. We summarize the simulation settings in Table 2.

In our simulation, two additional algorithms were used, as listed below.

1. Distributed computation offloading (DCO): The DCO algorithm exploits game theory for offloading decisions.
2. LE-based distributed computation offloading game (L&E DCO): The L&E DCO algorithm uses game theory based on latency and energy consumption [19].
3. Distributed clustering and computation offloading (DCCO): The proposed algorithm employs game theory for clustering and offloading decisions.

**Figure 3:**
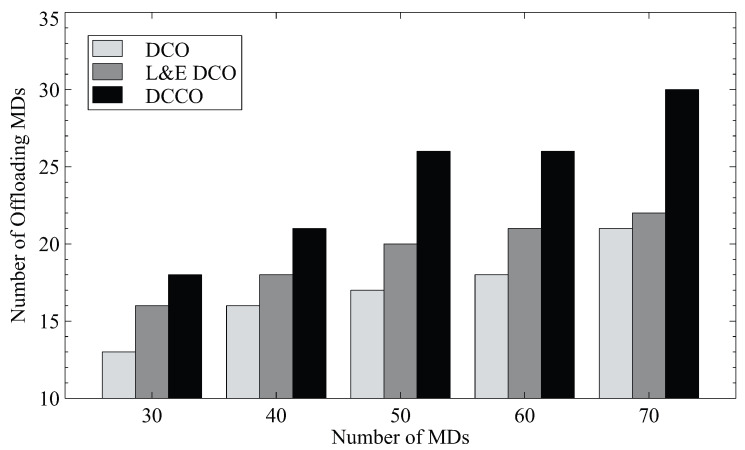
The numbers of offloading MDs for various algorithms.

First, we reveal the numbers of offloading MDs for the numbers of MDs in the different schemes in Figure 3. The scheme L&E DCO considers the ratio between edge and local computing to outperform the algorithm based on original game theory (DCO). The results show that it uses about 23% additional offloadings for 30 MDs as compared to DCO. However, our scheme, DCCO, can further increase the number of offloading MDs by 38%. It is apparent that DCCO outperforms both DCO and L&E DCO because of the additional MD clustering. The interference received by each MD is reduced, since the MDs in the same channel are eliminated. DCCO thus enables more MDs to offload tasks to the MEC server.

**Figure 4:**
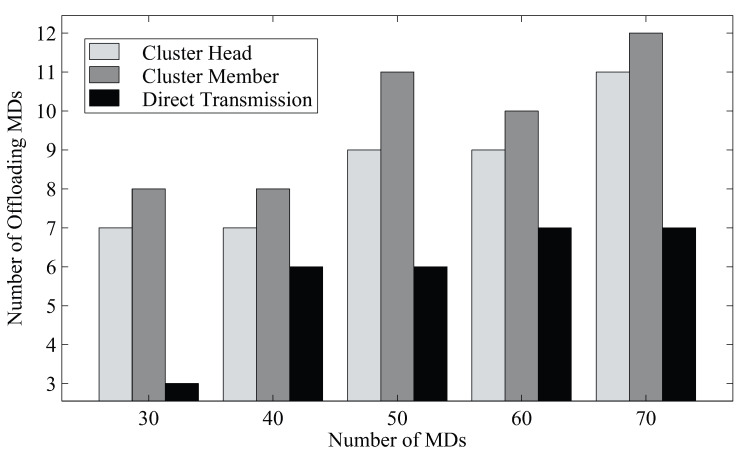
The numbers of cluster heads and members in each channel with 30 MDs.

We further show the numbers of cluster heads and members in Figure 4. The results show that the proposed algorithm can significantly increase the number of clustered MDs. When there are more MDs, more clusters are also generated to reduce the number of MDs directly communicating with the basestation.

**Figure 5:**
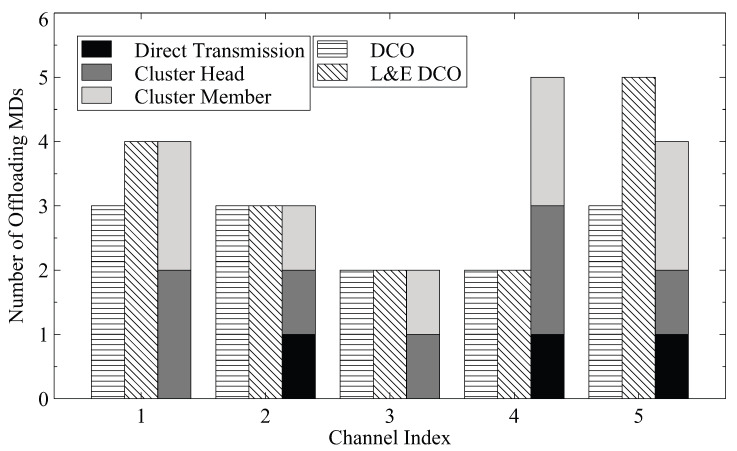
The distribution of 30 MDs in each channel.

We also show the number of MDs in each channel for the scenario with five channels and 30 MDs in Figure 5. The numbers of cluster heads and members for the proposed DCCO algorithm are also depicted. The results suggest that the additional offloading MDs can be achieved by MD clustering. Since the cluster members do not communicate with the basestation directly, the interference received by the mobile devices can be reduced to improve the transmission performance.

**Figure 6:**
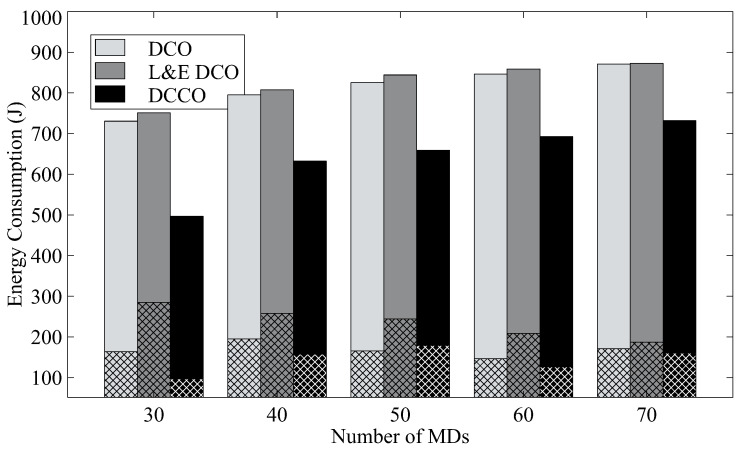
The average energy consumption with different algorithms.

Then, we indicate the energy usage in average for a mobile device to execute the task in Figure 6. The energy consumption includes that for both transmission and execution, where mesh bars denote the average energy consumption for data transmission. L&E DCO has similar energy consumption to DCO. In particular, L&E DCO consumes more energy for data transmission. As compared with the other two schemes, our scheme, DCCO, can reduce energy consumption by about 30% for 30 MDs by offloading more tasks to the MEC server.

**Figure 7:**
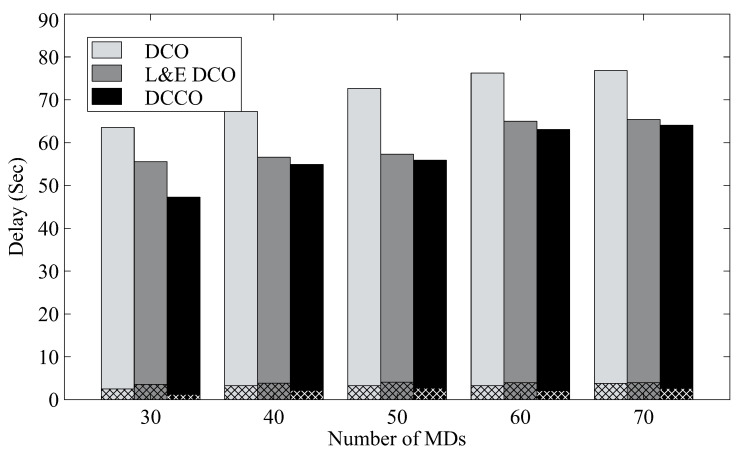
The average delay with different algorithms.

Figure 7 shows the average delay for finishing tasks from mobile devices for different numbers of MDs. The delay in the results also includes the latency for both transmission and execution, where the mesh bars depict average transmission delay. In the case of 30 MDs, it can be observed that the proposed algorithm has the shortest delay. With the DCCO algorithm, we can reduce response delay by about 20% as compared to DCO.

**Figure 8:**
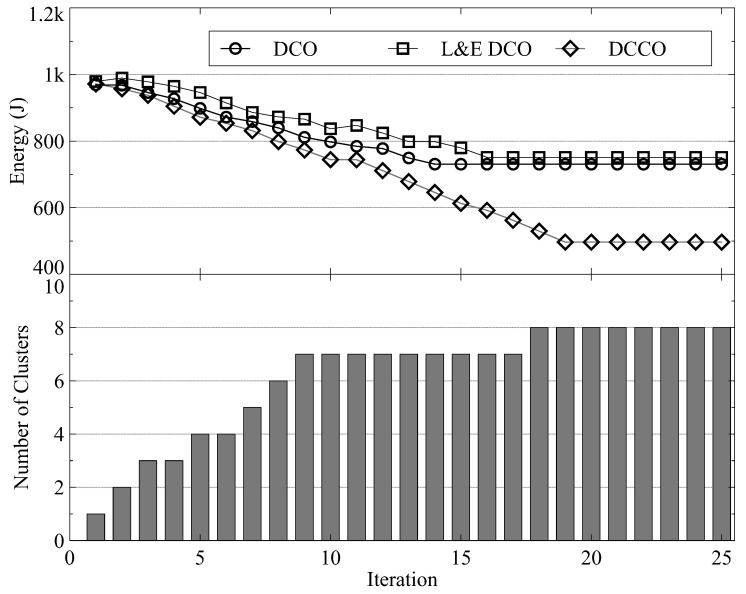
The convergence performance with 30 MDs.

**Figure 9:**
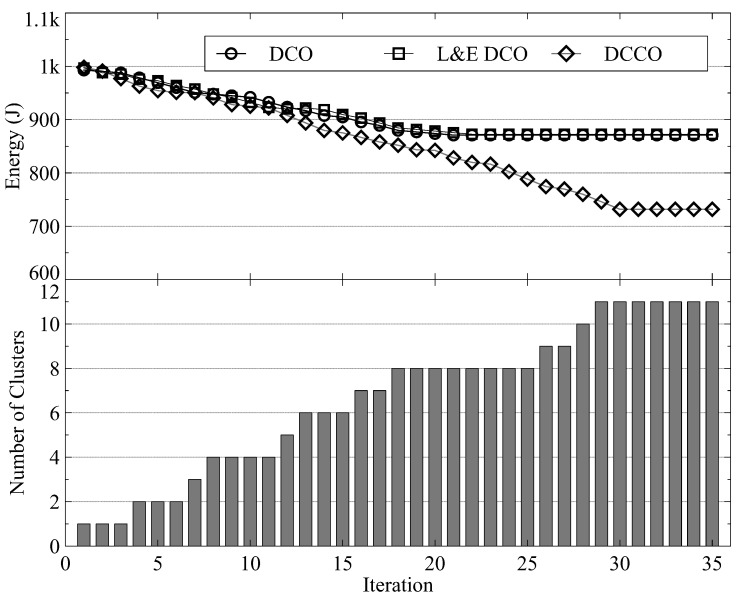
The convergence performance with 70 MDs.

Figure 8 and Figure 9 present the number of iterations for convergence with 30 and 70 MDs, respectively. In both figures, the upper half shows the energy consumption and the lower half shows the number of MD clusters. We observe that within 20 iterations, all schemes can reach Nash equilibrium. Although the proposed DCCO algorithm reaches the Nash equilibrium with more iterations than the other two algorithms, it also reduces the average energy consumption by increasing the number of clusters and offloading MDs. Figure 9 shows that about 35 iterations are required to reach Nash equilibrium. The results show that the convergence time is sublinearly related to the number of MDs. As a result, the proposed algorithm provides scalability for scenarios with numerous MDs.

## 7. Conclusions

In this work, we proposed an algorithm based on game theory to combine clustering and computation offloading to deal with increasing MDs in mobile edge computing. With MD clustering, the number of transmitting nodes in a channel can be reduced to improve the transmission rate because cluster members can forward data through their cluster heads. Accordingly, we formulated the overhead minimization problem as a competitive game and presented an algorithm for the clustering and computation offloading game. We also showed the existence of a Nash equilibrium for the game. In the performance evaluation, we showed that the proposed model for the distributed clustering and computation offloading game can achieve better efficiency of computation offloading than the previous game-theory-based schemes. With our algorithm, the number of offloaded tasks can be increased by up to 36% to lower the energy consumption of mobile devices by 30%. Our algorithm also shortens the latency of computation tasks by 20%. Moreover, our algorithm can effectively converge to yield feasible decisions of clustering and offloading. In our future work, we will attempt to improve the fairness of energy consumption among mobile devices, since mobile devices with poor channel quality may not be able to successfully offload their computation tasks.

## Figures and Tables

**Table 1:** Notation.

Symbol	Definition
*N*	The set of mobile devices
CL	The set of clusters
*M*	The set of channels
OFF	The offloading decisions of all MDs
$off_{n}$	The offloading decision of MD *n*
*C*	The clustering decisions of all MDs
$c_{n}$	The clustering decision of MD *n*
$J_{n}$	The computation task of MD *n*
$B_{n}$	The data size for $J_{n}$
$D_{n}$	The required CPU cycles for $J_{n}$
$T_{n}$	The computation time for $J_{n}$
$E_{n}$	The energy consumption for $J_{n}$
$p_{n}$	The transmission power of MD *n*
$r_{n}$	The transmission rate of MD *n*

**Table 2:** Simulation parameters.

Parameters	Value
Data size	5000 kbits
Number of CPU Cycles	$1000\times 10^{8}$ cycles
Number of Channels	5
Channel Bandwidth	5 MHz
Wi-Fi Channel Bandwidth	0.5 MHz
MEC Capacity	$10\times 10^{9}$ cycles/s
MD Capacity	$1.0\times 10^{9}$ cycles/s
MD Transmission Power	150 mWatts
MD Wi-Fi Power	50 mWatts
Noise	$10^{-10}$ mWatts
Path Loss Factor	4
Coverage	50 m

## Data Availability

Not applicable.
